# Notes on Preparations for the Sick

**Published:** 1899-09

**Authors:** 


### Notes on Preparations for the Sick.

The annual museum of the British Medical Association usually gives an excellent opportunity of learning what new things there are in food and physic; the recent display at Portsmouth was no exception to the rule. We propose to notice a few of the things which attracted our attention.

The **Somatose** group (Bayer & Co.), including Ferro-Somatose and Lacto-Somatose, which we have noticed on previous occasions (September, 1895, and March, 1899), appear to be establishing themselves as very useful manufactured foods in the convenient form of powders. Fairchild's **Peptogenic Milk Powder**, about which we said something last September, is also well known as a means for the production of normal mother's milk for infants. One of the most interesting series of food products has as its basis a powder prepared from milk, containing the milk proteids. It is called **Protene** (The Protene

COMPANY), and may be regarded as a dried milk from which the fat and sugar have been eliminated. It is obviously not a complete milk food, but is practically a pure proteid, which will keep for any length of time, can be added to or combined with a great variety of other foods, and cannot fail to be most valuable to the diabetic and the corpulent. A great variety of biscuits made with the protene flour as a basis provides suitable food for many different purposes and various tastes.

The use of a powder like protene, with the addition of cream and sugar, goes far towards the abolition of the morning and evening importation of so many tons of water inside the milk cans, which are such an annoyance diurnally at most of our railway stations. Why should not the milk be at once concentrated, and be kept in the house ready for use at any time by the mere addition of water? The ordinary condensed milks have not been as great a success as they deserve; possibly a milk powder would be more likely to attract the public fancy. Is not this the best solution of the difficulty as regards the prevention of tuberculosis by the sterilisation of milk? The abolition of the uncooked milk, and the sole use of the manufactured product, would reform the milk trade and be greatly to the advantage of the consumer. A milk powder, which may form the basis of a great variety of milk drinks, gives great scope for an indefinite production of products suitable to the needs of the invalid of the future.

The products of the AYLESBURY DAIRY COMPANY, HENRI NESTLÉ, and the ANGLO-SWISS MILK COMPANY are now well known to be perfectly trustworthy.

Several varieties of **Foods for Special Invalids** were exhibited by Messrs. CALLARD & Co., who provide not only for the diabetic but for the obese, the gouty and the rheumatic. They have also a special food to counteract constipation, a palatable, moist brown bread, a marmalade made with glycerine, and an infants' food described as non-farinaceous.

**Mellin's Food** products were much in evidence, including our old familiar friends the Mellin's Food Biscuits and the Cod Liver Oil Emulsion.

The **Beef Peptonoid** powder of Messrs. CARNRICK & Co., and the **Liquid Peptonoids** prepared from it, are beginning (though somewhat slowly) to be appreciated. The powder is efficiently sterilised during manufacture, and in the liquid preparation this aseptic condition is maintained by the addition of sufficient alcohol to preserve it. The combination with Coca is a palatable predigested fluid having much nutritive value.

The **Bovril** preparations are maintaining their reputation. One of the best of these is the raw **Meat Juice** expressed in the cold, and which is of the highest nutritive value.

Of the numerous preparations exhibited by Messrs. ALLEN AND HANBURYS the most interesting and novel were two combinations of Creasote: one, the phosphate, called **Phosote,** and the other, the tannophosphate, called **Taphosote.** They are best administered in capsules, each containing half a gramme. Six grammes have been given daily, and with a good result.

**Hommel's Hæmatogen** (NICOLAY & Co.) is a solution of Hæmoglobin, and should be an ideal blood-maker in cases where anæmia is a prominent feature, and more especially in cases where the inorganic preparations of iron are not proving satisfactory. In rickets, scrofula, and wasting diseases of childhood this fluid has given good results.

The **Hunyadi János Mineral Water** (ANDREAS SAXLEHNER) is too well known to require description. Its especial value at the present time is due to its presumed utility as an intestinal antiseptic in catarrhal gastro-enteritis of influenza type. It is recommended that it should be given freely, a pint on an empty stomach at the onset, and a glassful (!) on several consecutive mornings. The patient, purged of the influenza poison, is expected to escape all subsequent tendency to inflammatory complications, together with the adynamia and muscular debility.

